# Looking for a Straw in a Haystack by Bridging the Cracks Between Individual Judgments: Narrowing the Knowledge Gap To Anticipate Surprises by Transforming Risk Assessors’ Small Worlds Into Large Worlds

**DOI:** 10.1111/risa.70256

**Published:** 2026-04-23

**Authors:** James Derbyshire, Terje Aven

**Affiliations:** ^1^ Chester Business School Chester University Chester UK; ^2^ Department of Security, Economics and Planning University of Stavanger Stavanger Norway

**Keywords:** risk assessment, small‐world network, surprise, uncertainty

## Abstract

The world is constantly changing, yet a risk assessment is based on the knowledge available at one point in time. There will therefore be a gap between the range of possibilities known or conceivable to the assessor at that time and all the possibilities that could occur over infinite time. This will leave the door open to surprises. Anticipating surprises, therefore, requires the narrowing of this knowledge gap. We outline an approach to narrowing it that transforms risk assessors’ small‐world representations into a large‐world representation by configuring them into a small‐world network. Small‐world representations are individuals’ partial and subjective perspectives on an aspect of reality. What we call a large‐world representation integrates these small‐world representations. This transformation narrows the knowledge gap by integrating dispersed knowledge about, and intersecting alternative framings of, a focal risk, thereby dynamically updating the knowledge landscape underpinning its assessment. We use the 9/11 attack as an example. That surprise resulted from a failure to intersect the frames “suicide attack” and “hijacking,” meaning that the possibility of a “suicide hijacking” went unconsidered. Configuring risk assessors into a small‐world network would have increased the chance that these two frames would intersect in a risk assessment, thereby anticipating this surprise outcome. In sum, the approach we outline operationalizes the recently extended (*C*, *U*) risk‐assessment framework. It increases the chance that surprises are anticipated by enabling risk assessors to see as an integral whole what they could otherwise see only fragmentarily.

## 1 Introduction

Despite significant investment in risk assessments, governments, businesses, and other organizations are still regularly caught off guard by surprises. One reason for this is that, in seeking to anticipate surprises, risk assessors are not looking for the proverbial *needle* in a haystack. The task is much more complex than that. They are looking for a *straw* in a haystack that comprises a myriad other straws (Snowden 2024), many of which could plausibly be that which later emerges as crucial. Of course, once time and events have separated that one straw from the others, all can see it for what it is. Yet distinguishing it prospectively is a wholly different matter. That is the matter with which we are concerned herein.

Nevertheless, suffering the slings and arrows of outrageous fortune is far from inevitable. We are not destined forever to be corks bobbing in a sea of chance. We can anticipate what would otherwise be surprises, but this requires new tools and concepts tailored to that task. One such new concept is that of a “knowledge landscape,” as introduced by Derbyshire and Aven (2025) when setting out the extended (*C, U*) risk‐assessment framework (Aven 2016). A knowledge landscape comprises manifold knowledge elements, each representing a piece of evidence or information that a risk assessor has recognized as important to their belief in the possibility of an outcome and the severity of its consequences. This recognition is a matter of individual judgment, which is shaped by one's framing of the focal aspect of reality.

The question is: how do we bridge the cracks between individual judgments and intersect the framings from which they emerge, thereby ensuring the knowledge landscape is as complete as possible for all at the moment in time of a risk assessment? This is quite critical for avoiding surprises because knowledge elements are continuously generated, altered, or made obsolete by new developments that constantly change reality and the knowledge landscape that depicts it. The uncertainty this dynamism creates can only be overcome by bridging the cracks between individual judgments to achieve an integrated, holistic perspective on the focal risk.

In many contexts, this dynamism precludes closing the future by listing all its possibilities, which is an explicit requirement of many “traditional” risk‐analysis methods (Aven 2025). Cox (2020) describes these contexts as “open‐world settings,” but for reasons that will shortly become clear, we call them “large‐world settings.” Assessing risks in highly dynamic, large‐world settings is complex because risk assessments are based on the specific knowledge available at the time they are conducted. In large‐world settings, a risk assessment will therefore truncate the considered possibilities to those reflected in the knowledge available at that moment in time. This will inevitably exclude some possibilities whose potential only emerges later, leaving the door open to surprises.

This problem is *not* confined to periods of great crisis but persists in more mundane times (Pawson et al. 2011). The solution this paper provides is to dynamically update the knowledge landscape underpinning a risk assessment by transforming risk assessors’ individual small‐world representations into a large‐world representation. The concept of a small‐world representation is traceable to Leonard J. Savage's seminal text *The Foundations of Statistics* (Savage 1954). Small‐world representations are individuals’ partial and subjective perspectives on an aspect of reality (French 2015). A small‐world representation simplifies a decision to a choice from a fully listed set of actions, each of which is associated with a set of consequences (Feduzi et al. 2022). Which of these is actualized depends on which of a set of possible states of the world obtains (Feduzi et al. 2022).

Several authors have noted that using simplified small‐world representations becomes problematic in large‐world settings (Binmore 2008; Feduzi et al. 2022; Spiegelhalter 2024). Indeed, Savage (1954) recognized the possibility of large‐world settings in which uncertainty is unamenable to mitigation through creation of a small‐world representation (Feduzi et al. 2022). Accordingly, we proffer an alternative concept we call a large‐world representation. A large‐world representation integrates individuals’ partial and subjective perspectives on a focal aspect of reality (i.e. their small‐world representations), enabling the matter at hand to be seen as an integral whole.

In recognizing the possibility of large‐world settings in risk analysis, Aven (2025) calls on the field to adopt a broader perspective on risk that encompasses uncertainty in all its guises. Along those lines, Derbyshire and Aven (2025) extend the (*C*, *U*) framework, which recognizes the possibility of knowledge gaps and large‐world settings in a risk context. This paper complements that one by discussing how the extended (*C*, *U*) framework can be operationalized by transforming risk assessors’ small‐world representations into a large‐world representation using a “small‐world network.” (Watts and Strogatz 1998).

The “small world” referenced in this network's name was never intended by its originators to reference Savage (1954)'s small world of decision‐making. Accordingly, the two sets of literature in which they are discussed have, until now, developed separately. However, there is an important point of crossover between these similarly named concepts that appears to have escaped notice. Because of its idiosyncratic properties, a small‐world network can transform individual small‐world representations into a large‐world representation. Essentially, by integrating risk assessors’ small‐world representations and thereby intersecting their diverse framings of the focal risk, the small‐world network generates and diffuses new knowledge about that risk, dynamically updating its knowledge landscape into a large‐world representation.

However, if risk assessors frame information very differently, how can a common large‐world representation emerge from integrating their individual small‐world representations? Frames are sense‐making devices (Weick 1995) that mediate individuals’ interpretation of reality (Brugnach et al. 2008). Intersecting them is not a mere matter of integrating the knowledge elements on which they are based. It requires establishing some common ground, or a degree of agreement, about how that knowledge is interpreted. If risk assessors’ framings are diverse, how can there be any such agreement?

What we herein call a metaframe is a negotiated and co‐constructed communicative device produced through ongoing interaction (Brugnach et al. 2008). A metaframe suggests how information about a focal matter should be interpreted and transformed into knowledge (Dewulf et al. 2009). Essentially, by integrating its members’ small‐world representations and thereby intersecting their diverse framings of a focal risk, a small‐world network aids the co‐construction of a metaframe. The co‐construction of a metaframe is catalyzed by a small‐world network, and its construction helps ensure the network can collectively meet what Brugnach and Ingram (2012) call “the challenge of knowing and deciding together.”

Moreover, precisely because individual risk assessors will frame matters in diverse ways, intersecting their alternative framings enables “frame‐sensitive reasoning” (Bermúdez 2009, 2020, 2022). This involves decision‐makers being cognizant of how alternative framings of a focal matter foreground some aspects over others, thereby bringing some potential outcomes to prominence while rendering others obscured (Bermúdez 2020). Sensitivity to alternative framings is key to identifying possibilities that would otherwise fall into the cracks between individual judgments (Bermúdez 2020; Derbyshire 2024; Schum 1994). By intersecting risk assessors' diverse framings and stimulating frame‐sensitive reasoning, the small‐world network therefore helps bridge the cracks between individual judgments. This improves a network of risk assessors’ collective ability to anticipate surprises by enabling them to see, as an integral whole, what might otherwise be seen only fragmentarily.

In sum, then, this paper clarifies the epistemological origins of surprises, describes how to configure a network of risk assessors to improve their ability to anticipate, and operationalizes the extended (*C, U*) risk‐assessment framework by demonstrating how the knowledge gap it emphasizes (from which surprises arise) can be narrowed. Derbyshire and Aven (2025) used the 9/11 case to illustrate that framework when setting it out. That case is also employed herein because it provides the quintessential example of how failing to transform risk assessors’ small‐world representations into a large‐world representation leaves the door open to surprises.

The plan for the paper is as follows. Section two uses the extended (*C, U*) framework to conceptualize the knowledge gap and describe how it arises over time in large‐world settings. Section three illustrates the importance of narrowing this knowledge gap, using the surprise of the 9/11 case as an example. That section also offers a cautionary word on looking for a straw in a haystack. Section four outlines the small‐world network. Section five describes how the small‐world network narrows the knowledge gap. Section six describes how the small‐world network intersects risk assessors’ alternative framings and enables frame‐sensitive reasoning. Section seven contains concluding remarks.

## 2 Conceptualizing the Knowledge Gap

Surprises arise from knowledge gaps, which can be conceptualized using the extended (C, U) framework proposed by Derbyshire and Aven (2025). In that framework, risk is conceptualized by the two‐dimensional combination:

(1) $$\left(C,\;U\right)$$

where *C* is the consequence of the activity considered, and *U* is the associated uncertainty (i.e. what will *C* be?). A specific risk is then characterized by five components that are considered together:

(2) $$\left(C^{\prime },Q,K,\mathrm{UK},\;T\right)\;\;$$

where *C′* is a set of consequences (e.g. the number of fatalities), *Q* is an appropriate measure or description of uncertainty, *K* and *UK* are respectively the presently available (*K*) and presently unavailable (*UK*) knowledge about *C′* and *Q*. Together, *K* and *UK* comprise the knowledge landscape underpinning a risk assessment. *K* and *UK*―and, concomitantly, *C*′ and *Q*―change over time (*T*) as new developments occur. New developments generate, alter, or make obsolete the knowledge elements comprising the knowledge landscape (*K* and *UK*), updating both the knowledge landscape and the set of known consequences (*C′*) and their uncertainty (*Q*) over time (*T*).

Surprises result from the knowledge gap between *C′* in (2) and *C* in (1). The former represents the set of consequences known or conceivable to the risk assessors at the time of an assessment, as derived from the knowledge landscape available to them then. The latter represents all consequences that could occur over time, regardless of whether they are represented on or derivable from the knowledge landscape at the time of the risk assessment, and therefore, regardless of whether they are known or conceivable to the risk assessors at that time. In short, if the knowledge landscape is incomplete in an important way at the time of the assessment (e.g. important knowledge elements are missing from it), then a knowledge gap will emerge and *C′* in (2) will not correspond with *C* in (1). This will leave the door open to surprises.

Anticipating surprises therefore depends on narrowing the knowledge gap as much as possible, while accepting that it can never be entirely closed. It can never be entirely closed for two reasons. First, there will always be a delay in changes to reality occurring and their implications being recognized and incorporated into the knowledge landscape underpinning a risk assessment. Second, and relatedly, knowledge elements that later prove important may currently leave no empirical trace. These knowledge elements are unknown in a fundamental way—that is they are “unknown unknowns.”

However, unknown unknowns are not necessarily entirely unknowable (Derbyshire and Aven 2025). They might be inferred through the abductive reasoning (Snowden 2024) stimulated by intersecting risk assessors’ diverse “frames of reference” (Schum 1994, 228). This can increase the chance of perceiving novel knowledge elements and possibilities that might otherwise fall between the cracks of individual judgments (Schum 1994). Individual frames of reference are intersected by integrating risk assessors’ small‐world representations. However, as we outline next, it will remain challenging to anticipate surprises even when efforts are made to narrow the knowledge gap.

## 3 The Necessity but Insufficiency of Narrowing the Knowledge Gap by Integrating a Fragmented Knowledge Landscape

### 3.1 The Necessity of Integrating a Fragmented Knowledge Landscape: The Case of 9/11

Narrowing the knowledge gap first requires that new developments that change the knowledge landscape are promptly recognized by individual risk assessors (or risk‐assessing agencies) and then diffused rapidly, widely, and equally to all responsible for assessing the focal risk. This narrows the knowledge gap by transforming risk assessors’ individual small‐world representations into a large‐world representation. The latter is an integrated picture of the knowledge landscape, from which important yet absent knowledge elements can be inferred and novel possibilities derived. The surprise that was the 9/11 terrorist attack is a quintessential example of what happens when there is a failure to transform individuals’ small‐world representations into a large‐world representation.

de Bruijn (2006) details how the US Government's official 9/11 commission (National Commission 2004) attributed the failure to anticipate the 9/11 attack to the fact that, within the US intelligence community, “No single actor possesses all the relevant information…” even though “…an *integrated*, all‐source analysis of information is crucial…” (de Bruijn 2006, 269, emphasis added). Indeed, it is well documented that government agencies failed to adequately share knowledge before 9/11 (de Bruijn 2006; Glette‐Iversen and Aven 2021; Parker and Stern 2002). Posner (2005) corroborates de Bruijn (2006)'s interpretation of the 9/11 commission's official report and the fact that it attributes the failure to anticipate 9/11 to a “fragmentary” system for gathering and sharing knowledge.

As the subsequent sections of this paper demonstrate, configuring risk assessors into a small‐world network can help avoid knowledge fragmentation and its detrimental effect on the anticipation of surprises. Specifically, the small‐world network's idiosyncratic properties make it more likely that both individual risk assessors and the network as a whole will possess all the knowledge elements currently known to be relevant to a focal risk, regardless of where in the network they reside. But more than just that. The keyword in the quote from de Bruijn (2006) above has been emphasized in italics—*integrated*. An integrated analysis is crucial for anticipating surprises.

By integrating their individual small‐world representations into a large‐world representation, a small‐world network intersects risk assessors’ diverse framings of the focal risk, leading to the emergence of what we are calling a metaframe^1^. This can lead to a common understanding of how information about that risk should be interpreted and transformed into knowledge (Brugnach et al. 2008; Dewulf et al. 2009). This metaframe is a communicative device that helps bridge the cracks between individual judgments. There is some circularity here: a metaframe both helps produce and is itself a product of the emergence of a large‐world representation. We elaborate on this in the next subsection, below.

To understand what follows, it is important to note that, while the official 9/11 commission's report (National Commission 2004) correctly identified knowledge fragmentation as a primary cause of the failure to anticipate the 9/11 attack, it then proposed an intuitive yet misleading solution to this problem. Namely, the 9/11 commission's solution to the problem of a fragmented knowledge landscape was for increased centralization of intelligence gathering (Posner 2005). To implement this fix, it was recommended that a new role be created—an “intelligence czar” (Posner 2005). However, as will become clear in the discussion of the small‐world network that follows, the perhaps counterintuitive solution to knowledge fragmentation is *not* more control and centralization of knowledge gathering and analysis. Instead, as we discuss next, the solution is the opposite: a more distributed approach.

### 3.2 Looking for a Straw in a Haystack by Intersecting Alternative Frames of Reference

As the case of 9/11 testifies, integrating a fragmented knowledge landscape is essential to narrowing the knowledge gap and anticipating what would otherwise be surprises. However, while necessary, it is insufficient on its own. The history of surprises is littered with examples of someone discovering early indications of a potential surprise and yet being unable to direct key decision‐makers’ attention towards it (Snowden 2024). If events then actualize that surprise, the common refrain is “why didn't they just connect the dots?” (Snowden 2024). This is often accompanied by glib references to the need for a “holistic” perspective (Snowden 2024), as if integrating otherwise dispersed knowledge elements alone is sufficient to anticipate surprises. We want to be clear that transforming individual small‐world representations into a large‐world representation by integrating dispersed knowledge elements is necessary, but alone, insufficient to anticipate surprises. Snowden (2024) describes the reasons it is insufficient; we briefly summarize them here.

Imagine a knowledge landscape comprising four knowledge elements represented as differently colored dots. Six connections can be made between the dots^2^, and there are 64 possible patterns into which the dots could be configured^3^. Now increase the number of dots to 10. There are now more than 3 trillion possible patterns (Snowden 2024). Now consider that in a human system, there are usually far more than 10 “dots” (i.e. knowledge elements) of importance. With so many knowledge elements at play, risk assessors may be unable to see the woods for the trees. They will not merely be looking for the proverbial *needle* in a haystack. They will be looking for a *straw* in a haystack that comprises a myriad other straws, many of which could plausibly be that which later emerges as crucial (Snowden 2024).

Of course, when time and events distinguish that one straw from the others, it is easy to see it for what it is, and many may convince themselves that they “knew” it was that all along. Overconfidence in one's own judgment, as associated with hindsight bias, is quite common (Roese and Vohs 2012). In reality, we cannot “know” about the future in that sense. We can know about it in the sense of having a belief about it that is justified by knowledge. We cannot know the truth of those beliefs in the present and must wait for it to be revealed. In fact, the small‐world network we will shortly outline is about improving the justification of individual beliefs and their exchange and justification among risk assessors^4^.

Along similar lines, Snowden (2024) discusses experts’ “inattentional blindness,” which leads them to overlook apparent anomalies. Snowden (2024) argues cogently that simply improving information flows will not, by itself, overcome this problem. We would argue that the same is true of both integrating risk assessors' individual knowledge sets and centralizing knowledge gathering and analysis—the latter having been the solution recommended by the 9/11 Commission. Indeed, centralization is likely to worsen inattentional blindness rather than mitigate it. But then, so could integrating small‐world representations by simply aggregating their dispersed knowledge elements. The problem in both cases is complexity and is summed up by Cilliers (1998, 4) thus: “Each element [i.e. agent] in [a] system is ignorant of the behavior of the system as a whole, it responds only to information that is available to it locally…If each element ‘knew’ what was happening to the system as a whole, all of the complexity would have to be present in that element.”

However, we would argue that, if each element or agent in a system responds only to locally available knowledge, a key to anticipating surprises is to provide a way for the knowledge they each hold locally (i.e. within their small‐world representations) to be cross‐fertilized so that each has access to the “local” knowledge that resides more globally within the system. Not so that each can somehow possess a God's eye view of the whole system, which is impossible, but because this cross‐fertilization of knowledge will intersect their differing “frames of reference” (Schum 1994, 228; Snowden 2024). This intersection increases intersubjectivity and enables frame‐sensitive reasoning (Bermúdez 2009, 2020, 2022; Derbyshire 2024). The metaframe that may then emerge will enable the risk assessors to see both the wood and the trees by cutting through the thicket that obscures them.

When considering the properties of the small‐world network in the next section, the crucial point to note is that its effect is *not* merely to improve information and knowledge flows or integrate what would otherwise be dispersed knowledge elements. The small‐world network does do that, but not that alone. Moreover, the small‐world network's effect is certainly *not* one that centralizes knowledge gathering and analysis within any one godlike assessor or assessing agency. Instead, it is the small‐world network's effect on *both* improving information flows by integrating otherwise dispersed knowledge elements *and* its ability to intersect risk assessors’ diverse framings of a focal risk, which *together* assist in distinguishing that one crucial straw from the others in the haystack. Snowden (2024) suggests the key to anticipating surprises is a distributed sensemaking approach that employs diverse scanning networks that maximize diversity as much as expertise. For reasons that will become clear in the next section, a small‐world network does just that.

In setting out the extended (*C*, *U*) risk‐assessment framework, Derbyshire and Aven (2024) suggest “structured acts of imagination” to be essential for anticipating surprises. However, the official US Government report on the 9/11 attack stated that “Imagination is not a gift usually associated with bureaucracies…It is therefore crucial to find a way of routinizing, even bureaucratizing the exercise of imagination” (National Commission 2004; Weick 2005). When operationalized by configuring risk assessors into a small‐world network, the extended (*C*, *U*) risk‐assessment framework is a means by which to routinize the exercise of imagination. The small‐world network enables distributed sensemaking (Snowden 2024; Taarup‐Esbensen 2023) and stimulates the abductive reasoning associated with imagination by bridging the cracks between individual judgments. This enables what could be seen only fragmentarily to be seen as an integral whole. We now turn to describe the idiosyncratic properties of the small‐world network.

## 4 The Small‐World Network

### 4.1 A Brief Introduction to the Small‐World Network

The small‐world network (Watts and Strogatz 1998) formalizes Milgram (1967)'s seminal research on social connectedness. Milgram (1967) conducted an experiment in which packages were sent to select individuals in Omaha, Nebraska. These individuals were instructed to pass the packages on to someone living in Sharon, Massachusetts. However, they could only pass them on by sending them to personal acquaintances who would then forward them to their own personal acquaintances, and so on. A large percentage of these packages reached their targets, and Milgram (1967) recorded the number of steps or intermediaries required in each instance.

Milgram (1967) found that randomly selected people can be connected through an average of six acquaintances, and he coined this the “Small World network phenomenon,” leading to the popular phrase “six degrees of separation” (Derbyshire 2010). In what follows, we draw primarily on Cowan and Jonard (2004)'s description of the small‐world network because it explicitly considers its implications for knowledge diffusion—a matter of central importance to the small‐world network's ability to help narrow the knowledge gap and anticipate surprises within a risk assessment.

Cowan and Jonard (2004) formalize the small‐world network by first defining a basic “graph” or network as follows: let *I* = {1,…., *N*} denote a finite set of agents (i.e. network members). For any i,j∈I, define the binary variable χ(*i, j*) to take the value χ(*i, j*) = 1 if a connection exists between the agents *i* and *j*, and to take the value χ(*i, j*) = 0 if no connection between those agents exists. The network *G* = {χ(*i, j*); i,j∈I} is the list of all pairwise relationships between the agents in the network. The neighborhood of agent *i* is the set $\Gamma_{i}$ = {j∈I: χ(*i, j*) = 1}. A path in *G* connecting *i* and *j* is a set of pairwise relationships {($i,\;i_{1}$),…,($i_{k},\;j$)} such that χ($i,\;i_{1}$) = … = χ($i_{k},\;j$) = 1. The distance between *i* and *j* is defined as the shortest path between them, as represented by *d*(*i, j*).

**FIGURE 1:**
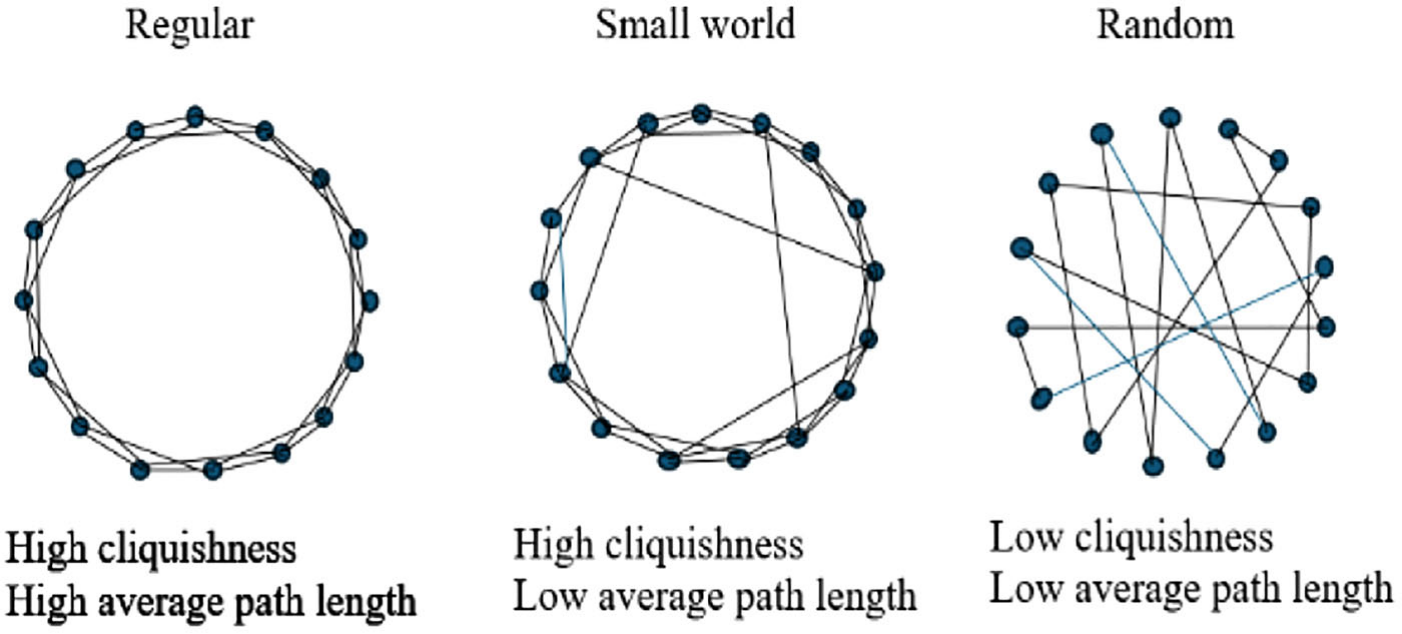
A regular, small world, and random network. Adapted from Cowan and Jonard (2004, 1560).

Having described the characteristics of a basic graph or network in this way, Cowan and Jonard (2004) then summarize Watts and Strogatz (1998)'s algorithm for the creation of a family of graphs or networks that lie between the two extremes of a nearest neighbor graph on a periodic lattice (i.e. a network in which all agents or network members are connected to their nearest neighbors) and a random graph with uniform degree^5^ (see the “Regular” and “Random” graphs in Figure 1, which represent these two extremes). The small‐world network (see the “Small world” graph in Figure 1) emerges in the transition between these two extremes, giving it specific properties that are very useful for the generation and diffusion of knowledge.

Watts and Strogatz (1998)'s algorithm for the emergence of a small‐world network goes as follows: take the regular graph in Figure 1 and sequentially consider each of its connections. With probability *p*, disconnect that connection from one of the agents it connects. Reconnect it instead to another agent chosen at random. By tuning *p* (i.e. by using alternative probabilities for this reconnecting), the graph's structure is varied from completely regular (where *p* = 0), through intermediate states (0 < *p* < 1), to completely disordered or random (where *p* = 1). This transition results in variation in the number of connections per agent. However, it maintains the average of *n* connections per agent and the total of Nn/2,∀p connections. The graph *G*(*n, p*) produced by this algorithm is the small‐world network in Figure 1.

### 4.2 The Small‐World Network's High Cliquishness Yet Low Average Path Length

The structural properties of the family of graphs or networks represented in Figure 1 are captured by the two concepts of “average path length” and “cliquishness” (Cowan and Jonard 2004). Cowan and Jonard (2004) define the cliquishness of a set of agents S⊆ *I* as the proportion of pairwise relationships in *S* over the total number of possible relationships:

(3) $$c\ell \;\left(S\right)=\frac{\Sigma_{i,\;j\in S}\;\chi \left(i,\;j\right)\;}{\#S\left(\#S-1\right)/2}$$

In a social network, cliquishness measures the proportion of an individual's contacts who are also friends or contacts of each other. By taking *S* to be the neighborhood of an agent in the network, cliquishness can be used to measure the network's local coherence in terms of “average neighborhood cliquishness”:

(4) $$C\;\left(\;p\right)=\Sigma_{i\in I\;}c\ell \left(\Gamma_{i}\right)/N$$

Average path length is defined as the average number of steps separating two randomly chosen agents in the network:

(5) $$L\;\left(\;p\right)=\Sigma_{i,\;j\in I\;}d\left(i,j\right)/N\left(N-1\right)/2$$

The average path length is the average number of other agents a focal agent must go through to reach a distant agent in the network with whom it is not directly connected.

Intuitively, one might expect cliquishness and average path length to be strongly positively correlated because, when cliquishness is high, agents’ connections are primarily local (i.e. with their nearest neighbors, as in the regular graph in Figure 1). So, the average path length will also be high. Where cliquishness is high, an agent in one neighborhood or clique must go through many intermediaries to reach a counterpart in a distant neighborhood or clique. And much of the time, cliquishness and average path length are indeed positively correlated, just as one might intuitively expect. However, there is an interval for *p* (the probability of a connection being reconnected) in which the small‐world network in Figure 1 emerges (Cowan and Jonard 2004), and cliquishness and average path length are no longer positively correlated.

**FIGURE 2:**
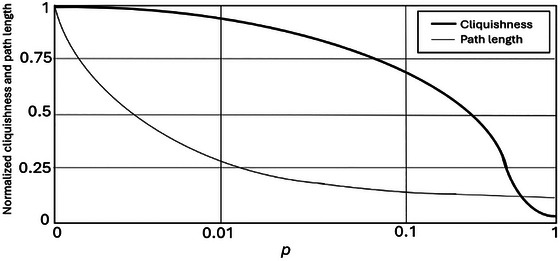
Average cliquishness and average path length as a function of *p*.

Figure 2 is adapted from Cowan and Jonard (2004) and shows the changes in average path length and clique size for different values of *p* in a graph with *N* = 500 vertices, where each agent has an average of *n* = 10 nearest neighbors. As evident in Figure 2, for the interval *p* ∈ [0.01, 0.1], “cliquishness and path length diverge, creating a small world region in the space of network structures” (Cowan and Jonard 2004, 1561) in which cliquishness is *high* yet average path length *low*. In short, when *p* (the probability of a connection being reconnected) lies between 1% and 10%, a small‐world network emerges that combines high cliquishness with low average path length. As we describe next, these two characteristics are crucial to the small‐world network's ability to narrow the knowledge gap and improve risk assessors’ ability to anticipate surprises by transforming their small‐world representations into large‐world representations.

## 5 Bridging the Cracks Between Individual Judgments to Transform Small‐World Representations Into a Large‐World Representation

To tie the above formalization of the small‐world network more explicitly to the task of narrowing the knowledge gap and anticipating surprises in a risk assessment, consider a (*C*, *U*)‐framework‐based risk assessment and its underlying knowledge landscape, which comprises multiple knowledge elements, whether known or unknown to an individual assessor (Derbyshire and Aven 2025). This knowledge landscape is initially fragmented because the knowledge elements that comprise it are currently dispersed among the knowledge sets of an extensive network of individual risk assessors or risk‐assessing agencies. Indeed, de Bruijn (2006) identifies at least 13 US Government agencies responsible for assessing risks related to terrorist attacks before 9/11, many of which were composed of additional internal sub‐agencies (de Bruijn 2006, Box 1, 269). These various agencies and sub‐agencies failed to adequately share intelligence (de Bruijn 2006; Glette‐Iversen and Aven 2021; Parker and Stern 2002; Posner 2005), leaving the knowledge landscape on which each assessed the possibility of an attack fragmented and incomplete.

The properties of the small‐world network make it highly efficient at integrating a knowledge landscape whose elements are fragmented across individual small‐world representations. However, these properties also make the small‐world network highly conducive to the generation and diffusion of *new* knowledge, not just the diffusion of existing knowledge. Knowledge elements that are not yet leaving any empirical trace may be inferred through abductive reasoning stimulated by intersecting risk assessors’ diverse framings (Schum 1994). Beyond merely integrating known but dispersed knowledge elements, this will narrow the knowledge gap by transforming what are presently unknowns into knowns. Together, these effects will transform individual risk assessors’ small‐world representations into a large‐world representation, thereby narrowing the knowledge gap by containing as many relevant knowledge elements as possible, whether knowns or unknowns. How the small‐world network enables this can be understood by comparing it with the regular and random networks that flank it in Figure 1.

The regular network in Figure 1 has high cliquishness because each network member in it is connected to its nearest neighbors. As a result, knowledge easily diffuses locally within the regular network. However, each network member's access to knowledge residing more distantly in the network (i.e. in distant network neighborhoods or cliques) is limited because of the regular network's high average path length. There are no network‐spanning connections that can serve as shortcuts to new knowledge elements residing in distant neighborhoods or cliques. It is necessary to go through many intermediaries to reach a network member in a distant neighborhood or clique. This creates a delay in receiving new knowledge from them (Gozzard et al. 2018). Moreover, because of the sheer number of intermediaries that must be traversed to receive new knowledge from them, there may also be an adverse effect on the accuracy of the knowledge received due to information entropy^6^ (Aprile et al. 2022). For example, note the number of other network members the bottommost agent in the regular network must go through to gain knowledge from the topmost.

In contrast, the random network in Figure 1 has a low average path length. So, in a random network, there is easy access to knowledge from distant neighborhoods or cliques. It is not necessary to go through many intermediaries to obtain this knowledge. The distinct knowledge sets of network members who would otherwise be socially distant from each other in the regular network are therefore intersected. However, the random network is also characterized by low cliquishness, meaning knowledge does not readily diffuse throughout the network. In this network, then, individuals’ small‐world representations will continue to differ in important ways. In the random network, it is difficult for a large‐world representation to emerge and become available to any one agent or to the network as a whole, compromising the ability to anticipate surprises either individually or collectively.

The small‐world network in Figure 1 resolves the tension between cliquishness and average path length in the regular and random networks. Because the members of a small‐world network simultaneously benefit from *high* cliquishness and *low* average path length,^7^ each can easily access new knowledge from every other member. At the same time, knowledge diffuses rapidly, widely and equally throughout the whole network. The amount and accuracy of knowledge transfer are therefore enhanced simultaneously (Aprile et al. 2022).

The small‐world network can simultaneously provide the double benefit of high cliquishness and low average path length because its connections follow a power‐law distribution (Zeng et al. 2025). This means that most network members have few connections, while a few have many. In the formal language of network theory, the small‐world network is characterized by a power‐law degree distribution (Zeng et al. 2025). The few network members with many connections ensure that all network members can reach each other easily. As a result, the small‐world network can be navigated efficiently, where navigability refers to the ability to route messages efficiently and in a decentralized manner (Fraigniaud and Giakkoupis 2009).

These characteristics first ensure that new knowledge diffuses rapidly, widely, and equally throughout the network, thereby transforming individual risk assessors’ small‐world representations into a large‐world representation, enabling the knowledge landscape to be seen as an integral whole. Second, integrating risk assessors’ small‐world representations in this way stimulates abductive reasoning by intersecting the diverse framings of the focal risk across individual risk assessors. This can help generate new knowledge that transforms unknowns into knowns (Schum 1994). We further explore these important effects in the final section.

## 6 Narrowing the Knowledge Gap by Connecting the Dots and Stimulating Abductive Reasoning

To understand how configuring risk assessors into a small‐world network can assist not merely in integrating existing knowledge but in generating new knowledge that transforms unknowns into knowns, consider once more the case of 9/11. We have stated that the 9/11 case is a quintessential example of a surprise caused by the failure to transform individual small‐world representations into a large‐world representation. That is because many of the knowledge elements needed to anticipate the 9/11 attack were known but dispersed across the small‐world representations of individual risk assessors operating in different government agencies. These agencies failed to “connect the dots” (Glette‐Iversen and Aven 2021, 10) by integrating these knowledge elements into a large‐world representation.

However, the problem was not so much the failure to integrate relevant knowledge elements that were already known, but rather, the fact that this failure meant that the “frames of reference” (Schum 1994, 228) suicide attack and hijacking—both of which were separately part of individual risk assessors’ small‐world representations—were not intersected. This meant that a possibility that resided within the cracks between these frames—that is that of a suicide hijacking—went unrecognized (Weick 2005). That failure contributed to exactly the surprise outcome of a suicide hijacking (Weick 2005). The failure to connect the dots meant that an important possibility lurking between them went unrecognized. It is not so much the connection of the dots but the intersection of frames to which it leads that matters.

Weick (2005) discusses the need for imagination in anticipating such surprises. According to Weick (2005), imagination is the “ability to conceive of something, seen only fragmentarily or superficially, as a complete, perfected, and integral whole” (Weick 2005, 427). Configuring risk assessors into a small‐world network enables a knowledge landscape that could otherwise be seen only fragmentarily to be seen as an integral whole. This is not simply because a small‐world network integrates risk assessors’ individual small‐world representations into a large‐world representation by aggregating their dispersed knowledge elements. More importantly, doing so makes it more likely that the network's members will recognize important but presently absent knowledge elements and possibilities that lie in the gaps between those knowledge elements.

This needs to be unpacked more. From the perspective of an individual risk assessor, new questions or observations—stimulated by integrating new knowledge elements from socially distant sources into her small‐world representation—put her at an intersection with a new and alternative “frame of reference” (Schum 1994, 228). By integrating this frame with the one she already possesses, this can lead to “flashes of insight” (Schum 1994, 474) characteristic of abductive reasoning, setting her thinking on a different path and leading to consideration of wholly new possibilities. Moreover, the same effect on other members of the network can lead to the emergence of a metaframe that provides the common ground needed to interpret new information in a sufficiently similar way as to enable collective intelligence and distributed sensemaking.

The point to be emphasized is that, rather than merely aggregating dispersed knowledge elements and hoping for the best, configuring risk assessors into a small‐world network increases intersubjectivity and frame‐sensitive reasoning by intersecting the assessors’ diverse framings of the focal risk. Like triggering a gestalt switch, intersecting risk assessors’ diverse framings can enable patterns to stand out that would otherwise remain submerged and obscured within the many knowledge elements on a knowledge landscape and the thousands, if not millions, of possibilities (i.e. plausible scenarios) they could form through recombination. Configuring risk assessors into a small‐world network, therefore, helps meet “the challenge of knowing and deciding together” (Brugnach and Ingram 2012). It helps find that one straw obscured in the haystack that later proves crucial by bridging the cracks between individual judgments, thereby narrowing the knowledge gap and anticipating surprises.

## 7 Conclusion

The world is constantly changing, yet a risk assessment is based on the knowledge available to risk assessors at the single point in time when an assessment occurs. There is, therefore, likely to be a knowledge gap between the range of possibilities conceivable to the assessors at the time of their assessment and the full range of possibilities that could occur over infinite time. Considering this, it is unsurprising that surprises regularly catch out governments, businesses and other organizations, despite their significant investment in risk assessments.

However, suffering the slings and arrows of outrageous fortune is far from inevitable. We can improve our ability to anticipate surprises by narrowing the gap between the possibilities assessors can conceive when making their assessment and the full range of possibilities that could occur over time. Large‐world settings may include novel and emergent possibilities that leave little or no empirical trace at the time of the assessment. However, for the reasons outlined herein, narrowing this knowledge gap requires more than merely integrating dispersed knowledge elements. That is necessary, but is alone insufficient for anticipating surprises. The requirement is to generate new knowledge, not merely aggregate that which already exists.

Simply providing the pieces of a one‐thousand‐piece puzzle does not by itself guarantee that they will be combined into the correct pattern, especially if some of the pieces are missing, and even more so if the bounds of the puzzle and the pieces comprising it are both changing over time as one puts it together. Moreover, when there are so many pieces, simply reshuffling them into different combinations and orders, in the vague hope of finding the correct pattern, will not help. Furthermore, while they must be pieced together correctly, when it comes to anticipating surprises in large‐world settings, either there is no picture on the box to guide their assembly, or the picture is very blurry and changing, providing limited guidance. This makes solving the puzzle difficult, as the continued occurrence of surprises testifies.

Finding the one straw in the haystack that later proves crucial requires more than simply improving knowledge flows and integrating otherwise dispersed knowledge elements because the straws all look the same when they are in the haystack. In prospection, even relatively bounded aspects of reality present many possibilities that are all logically consistent and seemingly plausible. Moreover, the number of possibilities increases exponentially as the number of knowledge elements increases.

Fortunately, by transforming risk assessors’ small‐world representations into a large‐world representation, the approach we have outlined does more than merely integrate otherwise dispersed knowledge elements. Its effect is to increase intersubjectivity and frame‐sensitive reasoning, thereby enabling the abductive reasoning associated with acts of imagination. This leads to the generation of new knowledge, not merely the integration of that which already exists. The approach enables what might otherwise be seen only fragmentarily to be seen holistically. Rather than merely connecting the dots, stimulating abductive reasoning generates new dots by interpolating between those already there, thereby filling knowledge gaps and transforming unknowns into knowns. From the more holistic picture available to risk assessors and from the intersection of their alternative framings, like the triggering of a gestalt switch, a pattern may emerge that could not otherwise be seen.

## Conflicts of Interest

The authors declare no conflicts of interest.

## Endnotes

1 There is some crossover here with what Bermúdez (2020) calls “meta‐awareness,” and with the more general concept known as “metacognition” that is widely used in cognitive psychology, both of which might be understood as thinking about thinking, or being aware of how one is thinking about a focal matter and how that affects the thoughts one is having about it. Bermúdez (2020, 247) contrasts two states of meta‐awareness: a first‐order state of thinking about *p* and a second‐order state of thinking about how one is thinking about *p* and how that is shaping one's judgment of it.

2 Imagine the four dots as the four corners of a square, which are then joined by two horizontal and two vertical connections around the edges, and two diagonal connections across the middle.

3 Imagine the four dots as each having a different color that distinguishes them. There are 64 differently ordered colored patterns possible.

4 In this respect, the method we outline herein, based on the small‐world network, has some crossover with the Delphi method for group‐based judgment (Belton et al. 2019). The Delphi method bridges between individuals’ judgments and enables frame‐sensitive reasoning (of a type), but in a very structured and explicit way: participants independently arrive at an initial estimate of a quantity, are provided with the reasoning behind alternative estimates from other participants, and then asked to reconsider their initial estimate. We do not explore this crossover further herein.

5 In a graph or network with “uniform degree” every agent—or in the formal language of graph theory, every “node” or “vertex”—in the graph has the same number of connections (or “edges”). A connection or edge is a link that connects two agents or network members.

6 Or to give information entropy its alternative and perhaps more technically correct name, Shannon entropy (Mishra and Ayyub 2019).

7 In having these simultaneous characteristics of high cliquishness and low average path length, there is some crossover between the small‐world network and so‐called “expander graphs.” The latter share the characteristic of being simultaneously sparse yet highly connected (Hoory et al. 2006).
